# Immunomodulatory Nanosystems: Advanced Delivery Tools for Treating Chronic Wounds

**DOI:** 10.34133/research.0198

**Published:** 2023-07-14

**Authors:** Xiangyu Chu, Yuan Xiong, Samuel Knoedler, Li Lu, Adriana C. Panayi, Michael Alfertshofer, Dongsheng Jiang, Yuval Rinkevich, Ze Lin, Zhiming Zhao, Guandong Dai, Bobin Mi, Guohui Liu

**Affiliations:** ^1^ Department of Orthopedics, Union Hospital, Tongji Medical College, Huazhong University of Science and Technology, Wuhan 430022, China.; ^2^ Hubei Province Key Laboratory of Oral and Maxillofacial Development and Regeneration, Wuhan 430022, China.; ^3^ Division of Plastic Surgery, Brigham and Women’s Hospital, Harvard Medical School, Boston, MA 02152, USA.; ^4^ Institute of Regenerative Biology and Medicine, Helmholtz Zentrum München, Max-Lebsche-Platz 31, 81377 Munich, Germany.; ^5^ Department of Hand, Plastic and Reconstructive Surgery, Microsurgery, Burn Center, BG Trauma Center Ludwigshafen, University of Heidelberg, Ludwig-Guttmann-Strasse 13, 67071 Ludwigshafen/Rhine, Germany.; ^6^ Division of Hand, Plastic and Aesthetic Surgery, Ludwig - Maximilian University Munich, Munich, Germany.; ^7^ Department of Orthopedics, Suizhou Hospital, Hubei University of Medicine, Suizhou 441300, China.; ^8^ Pingshan District People’s Hospital of Shenzhen, Pingshan General Hospital of Southern Medical University, Shenzhen, Guangdong 518118, China.

## Abstract

The increasingly aging society led to a rise in the prevalence of chronic wounds (CWs), posing a significant burden to public health on a global scale. One of the key features of CWs is the presence of a maladjusted immune microenvironment characterized by persistent and excessive (hyper)inflammation. A variety of immunomodulatory therapies have been proposed to address this condition. Yet, to date, current delivery systems for immunomodulatory therapy remain inadequate and lack efficiency. This highlights the need for new therapeutic delivery systems, such as nanosystems, to manage the pathological inflammatory imbalance and, ultimately, improve the treatment outcomes of CWs. While a plethora of immunomodulatory nanosystems modifying the immune microenvironment of CWs have shown promising therapeutic effects, the literature on the intersection of immunomodulatory nanosystems and CWs remains relatively scarce. Therefore, this review aims to provide a comprehensive overview of the pathogenesis and characteristics of the immune microenvironment in CWs, discuss important advancements in our understanding of CW healing, and delineate the versatility and applicability of immunomodulatory nanosystems-based therapies in the therapeutic management of CWs. In addition, we herein also shed light on the main challenges and future perspectives in this rapidly evolving research field.

## Introduction

Chronic wounds (CWs) pose a substantial burden for patients and their families, often arising as secondary complications associated with increasing age, obesity, diabetes, or vasculature insuficiency [1]. The prevalence of CWs has increased rapidly over the past decade, translating to a rising incidence of CW-related amputations or sepsis [2]. Notably, CWs may persist for years or even a lifetime, causing severe psychological and physical distress and imposing substantial financial burden on both patients and the healthcare system. In the United States alone, it is estimated that up to 4.5 million people suffer from CWs, resulting in enormous healthcare costs [3]. Pathophysiologically, CWs are typically characterized by multiple-drug-resistant bacterial infections, immune disorders, angiopathy, neuropathy, and elevated oxidative stress levels [4,5]. Due to the complex pathogenesis of CWs, traditional therapeutic strategies, including antibiotic therapy, surgical debridement, skin transplantation, and the application of wound dressings, have failed to achieve the required effectiveness. In addition, the prolonged duration of CW treatments carries the risk of physical and psychological sequelae for patients [6]. Therefore, there is an urgent need for the development of effective, time-efficient, and minimally painful therapies for CWs.

CWs are marked by a prolonged inflammation resulting in the impairment of tissue regeneration and repair [7]. Thus, the aberrant immune microenvironment of CWs has gained increasing attention in the research of wound therapy [8]. In contrast to acute wounds, CWs are characterized by excessive immune cell infiltration, overproduction and release of inflammatory cytokines, compromised blood supply, and elevated levels of oxidative stress in CWs, all of which prolong inflammation and disrupt the coordinated healing process [9]. To facilitate the curative healing process of CWs, immunomodulatory therapy is required to interrupt such detrimental positive feedback loop. In this context, nanosystems featuring excellent physicochemical properties, efficient drug-loading capacity, and favorable biocompatibility are emerging to modulate the pathogenic immune microenvironment of CWs with therapeutic intent [10].

**Fig. 1.**
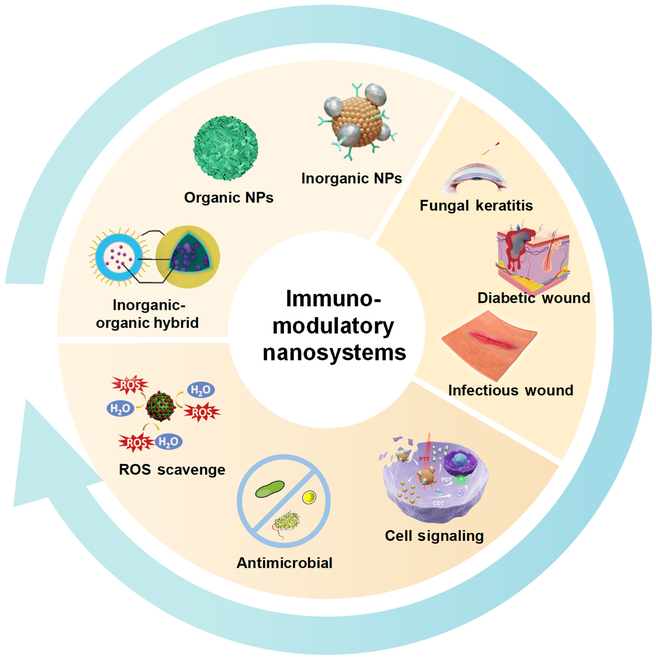
Outline of the categories, mechanisms, and applications of nanosystems in CWs. Immunomodulatory nanosystems have garnered considerable interest due to their ability to modify the immune microenvironment of CWs and promote wound healing without the drawbacks associated with systemic side effects. These nanosystems possess favorable properties, such as adjustable size, variable charge, and a high surface-to-volume ratio. These characteristics make them well suited for drug delivery systems, enabling the encapsulation of bioactive factors and molecules with superior pharmacokinetic and pharmacodynamic profiles. Reproduced with permission from [117,130,135]. Copyright (2022, 2021, 2022) American Chemical Society.

In recent decades, advancements in nanotechnology have enabled the design and utilization of nanosystems for the treatment of CWs (Fig. 1) [11,12]. Immunomodulatory nanosystems are of particular interest, offering the capability to modify the immune microenvironment of CWs and, thus, promote wound healing without the drawbacks of systemic side effects. Immunomodulatory nanosystems exhibit favorable properties (i.e., adjustable size, variable charge, and high surface-to-volume ratio), rendering them well suitable for drug-delivery systems and allowing for encapsulation of bioactive factors and molecules with superior pharmacokinetic and pharmacodynamic profiles [13]. Recently, seminal reviews have highlighted the key advances and unique properties of immunomodulatory nanosystems [14,15]. For example, Feng et al. [14] have discussed the emerging utilization of immunomodulatory nanosystems in a variety of diseases, including cancers and infectious diseases, through local immunostimulation and/or immunosuppression. However, to date, there is a paucity of comprehensive reviews specifically focusing on nanosystems-based drug delivery and their underlying mechanisms of action in CW treatment.

Herein, we aim to delineate the main processes of CW healing, with a specific emphasis on the immune microenvironment of CWs. We provide a summary of nanosystems-based strategies aimed at modulating the immune microenvironment and offer valuable insights for the design of more effective immunomodulatory nanosystems for CWs. Furthermore, we address the current challenges and future perspectives related to immunomodulatory nanosystems, thereby paving the way for their translation into clinical practice.

## Pathogenic Characteristics of CWs

**Fig. 2.**
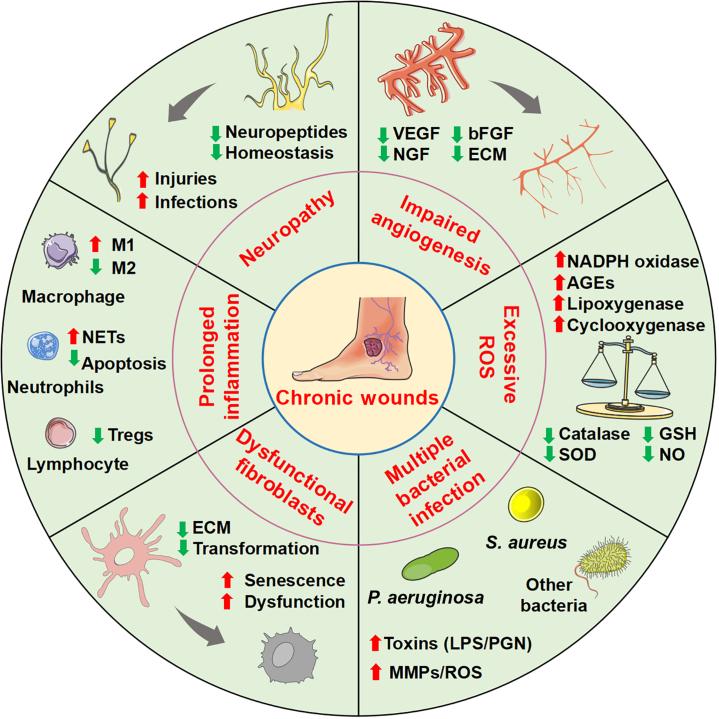
Pathogenic characteristics of CWs. Prolonged inflammation caused by neutrophils infilitration, reduced Tregs, and imblanced M1/M2. Excessive ROS in CWs can cause the activation of various signaling pathways that lead to vascular endothelial cell damage and impaired angiogenesis. Senescent/dysfunctional fibroblasts can reduce myofibroblast transformation and ECM secretion. The decrease in VEGF, nerve growth factor (NGF), bFGF, and other angiogenesis-stimulating growth factors contribute to impaired wound angiogenesis. Skin sensory nerves play a regulatory role in inflammation, immunity, cell proliferation, and vascular proliferation and participate in wound healing. Various bacterial species, including *P. aeruginosa*, *S. aureus*, and other bacteria lead to wound infection, toxins, and MMPs, which impair re-epithelialization of wound repair cells and consequently delay wound healing. NADPH, nicotinamide adenine dinucleotide phosphate; AGEs, advanced glycation end products; SOD, superoxide dismutase; GSH, glutathione; PGN, peptidoglycan.

CWs are prolonged nonhealing tissue injuries that persist on the body surface for more than 3 months, impeding the re-establishment of anatomical and functional integrity through normal reparative processes [16]. The etiology of chronic trauma encompasses a plethora of factors, including diabetic ulcers, pressure ulcers, and arteriovenous ulcers. Therefore, there is an urgent need to elucidate the pathogenesis and pathological features of CWs while exploring effective treatment strategies. The pathological mechanisms of CWs is complex, diverse (caused by both systemic and local factors), and incompletely understood [17]. The underlying mechanisms appear to be multifaceted and are believed to be primarily driven by the following aspects: exaggerated and persistent inflammatory response, tissue damage caused by the accumulation of reactive oxygen species (ROS), dysfunctional fibroblasts, vascular lesions, neuropathy, and multiple bacterial infections (Fig. 2).

### Excessive and prolonged inflammatory response

An excessive and prolonged inflammatory response is an important pathogenetic mechanism underlying the development of CWs. Cutaneous wound healing typically follows a dynamic process consisting of 4 distinct yet overlapping phases, namely: hemostasis, inflammation, proliferation, and tissue remodeling [18]. While this healing process is well coordinated in acute wounds, the repair mechanisms in CWs become disrupted, often preventing the progression beyond the inflammatory phase, subsequently hindering cell proliferation and matrix accumulation at sites of injury, ultimately leading to delayed wound healing [19].

During the inflammatory response, the number of neutrophils, M2 macrophages, and proteases correlates with the severity of the wound. Neutrophils exhibit hyperactivity, abnormal apoptosis, and excessive production of neutrophil extracellular traps (NETs), which can impair wound healing [20]. Neutrophils secrete a multitude of cytokines, including tumor necrosis factor-α (TNF-α), interleukin-1β (IL-1β), and IL-6, to enhance the inflammatory response. Neutrophils can also release various antimicrobial substances, including cationic peptides and proteases for wound debridement [21]. However, excessive neutrophil activity and abnormal apoptosis result in abnormally high levels of these substances beyond the normal reparative range. Macrophages infiltrate the wound site in increased numbers in CWs, featuring abnormal phenotypic transformation and impaired efferocytosis, which disrupt the healthy wound healing process [22]. Proinflammatory macrophages secrete inflammatory mediators, including TNF-α, IL-17, IL-1β, ROS, and inducible nitric oxide synthase. In addition, abnormal phenotypic transformation and impaired efferocytosis result in negative regulation of wound healing. Increased TNF-α levels lead to excessive secretion of matrix metalloprotein-1 (MMP-1) and MMP-3 while inhibiting the secretion of tissue inhibitor of metalloproteinase-1. Elevated levels of IL-1β have a similar effect as TNF-α, prolonging the expression of each other, thereby delaying the inflammatory response in CWs. The increased secretion of MMPs contributes to excessive hydrolysis of extracellular matrix (ECM) in CWs [23]. ECM generally serves as a scaffold for cell migration and wound closure, and its absence hinders the wound healing process. High MMP-9 activity is considered to sustain the wound in an inflammatory state and is often indicative of poor wound healing outcomes [24].

In summary, excessive numbers of neutrophils and macrophages in CWs lead to altered cellular responses, resulting in a refractory microenvironment characterized by abnormal growth factors, imbalanced protein degradation, and prolonged inflammation. As infection and inflammation persist in CWs, the wound remains trapped in a cycle of infection, inflammation, and inadequate repair, ultimately exhibiting chronic delayed wound healing. Kwak et al. [25] utilized hydrolytically degradable poly (ethylene glycol) hydrogel system to deliver M2-Exos, which contains putative key regulators driving macrophage polarization, exhibiting therapeutic effects in an animal model for cutaneous wound healing including rapid wound closure and increased healing quality. Zhang et al. [26] developed PLGA@IL-8 nanoparticles-loaded acellular dermal matrix as a delivery system for exogenous mesenchymal stem cells (MSCs) in diabetic wound healing, which indicated a potential therapeutic dressing that may contribute to the therapy of diabetic wounds by anti-inflammation, capillary construction, and collagen deposition. Therefore, regulating the local inflammatory microenvironment is an important method to promote the healing of CWs.

### ROS damage

Elevated levels of ROS at the wound site also contribute to the pathogenesis of CWs. Redox balance is crucial for effective wound healing, with ROS operating as key regulators and playing a key role in tissue regeneration. Accordingly, the successful healing and regeneration of injured tissue depend on the optimal balance between the beneficial and detrimental effects of ROS [27]. Under normal circumstances, low levels of ROS are necessary to counteract external injuries. ROS aid in pathogen destruction within the wound and serve a protective role in the host defense system by dampening phagocytosis. Additionally, ROS act as redox messengers for various immune and nonlymphoid cells, thus regulating angiogenesis [28]. The massive infiltration of inflammatory cells in CWs leads to excessive ROS production, which interferes with the balance between oxidation and antioxidant mechanisms. This tissue oxidation imbalance results in damage to DNA, proteins, lipids, and cells, further triggering cellular senescence, and uncontrolled inflammation [27].

ROS can aggravate the stress response of functional cells, particularly evident through increased activity of signaling pathways involved in the secretion of proinflammatory cytokines, chemokines, and MMPs within the injured tissues. ROS can also directly activate proteases (i.e., MMPs and serine protease) and inactivate protease inhibitors, degrade key growth factors such as platelet-derived growth factor and transforming growth factor-β1 (TGF-β1), resulting in increased protein hydrolysis [29]. This further exacerbates ECM degradation, which is closely associated with impaired wound healing [30]. In addition, ROS can directly impair the functionality of healthy MSCs, resulting in decreased proliferation, migration and adhesion, and induction of apoptosis [31]. The dysfunction of MSCs hampers tissue re-epithelialization and granulation tissue formation, negatively affecting wound healing. Finally, local senescent cells within the wound exhibit reduced responsiveness to normal wound healing stimulation and occupy limited space. Senescent fibroblasts also produce elevated levels of proteases (such as MMP-2, MMP-3, and MMP-9) while exhibiting a decrease in protease inhibitors, further prolonging the wound healing process [32]. Thus, efforts to remove excessive ROS from wounds may facilitate wound healing. For example, Wu et al. [33] successfully synthesized a highly versatile nanocomposite tissue adhesive that scavenges ROS by immobilizing ultrasmall ceria nanocrystals on the surface of uniform mesoporous silica nanoparticles (MSN). This innovative approach was found to promote wound repair and regeneration. Therefore, the utilization of biomaterials to regulate ROS levels and control oxidative damage in injured tissues holds therapeutic potential in the management of CW healing without disrupting healthy physiological processes in the human organism.

### Dysfunctional fibroblasts

Skin fibroblasts play a pivotal role in the proliferation and remodeling stages of the wound healing program through activities such as cell proliferation, myofibroblast transformation, and secretion of the ECM [34,35]. In addition, fibroblasts actively contribute to the inflammatory phase by engaging in bidirectional interactions with immune cells infiltrating the wound [36]. Chronic nonhealing wounds are typically characterized by the inability of the repair process to progress from the inflammatory phase to the proliferation phase. This impairment may also be due to maldifferentiation/function of fibroblasts.

There is a mounting body of evidence pointing to a significance for the subcutaneous fascia in successful wound healing [37,38]. The fascia is a viscoelastic layer of connective tissue situated beneath the dermis. More specifically, the mobilization of fascial connective tissue and cells was found to facilitate the transportation of essential cellular and matrix components necessary for the initiation of the wound repair process. Throughout the healthy wound healing process, fascial fibroblasts undergo a series of differentiation steps, starting from a homeostatic state characterized by progenitors expressing CD201 that then transition into an inflammatory state marked by podoplanin expression. Subsequently, podoplanin-positive proinflammatory fibroblasts progress into a proto-myofibroblast state marked by phosphorylation of activator of transcription 3 expression and finally reach a myofibroblast state as identified by α-smooth muscle actin expression. These transitions enable fascial fibroblasts to complete a wide array of crucial functions ranging from proliferation through ECM production to tissue remodeling [39]. However, in CWs, excessive and prolonged inflammatory responses hinder the transition of fascial fibroblasts to the myofibroblast state. This phenomenon is commonly observed in conditions such as diabetic foot ulcers, where fascial fibroblasts persist in a proinflammatory state instead of progressing to the desired myofibroblast state [6,40]. Furthermore, the hostile proinflammatory (micro)environment in CWs may adversely affect intercellular adhesion and communication between fascial fibroblasts. As a result, key components involved in fibroblast cell-cell communication, including N-cadherin-based adherens junctions [41], Connexin43-based gap junctions [42], and the associated p120 catenin [43], may be suppressed. Notably, these cellular interactions are required for the coordinated collective migration of fascial fibroblasts at a supracellular level [41], leading to normal wound contraction and wound closure after skin wounding.

In addition, the excessive production of ROS contributes to increased fibroblast senescence, which is a pathological mechanism seen in chronic venous leg ulcers [44]. In these ulcers, fibroblasts exhibit an elevated expression of senescent markers, such as p16 (Ink4a) and p21 (Cip1/Waf1), both of which have been shown to impede fibroblast proliferation, delay granulation tissue formation, and impair wound healing [45,46]. In addition, accumulated senescent fibroblasts release proinflammatory and tissue-degrading factors known as senescence-associated secretory factors characterized by IL-1β, IL-6, and IL-8. These factors, in turn, contribute to the establishment of a chronic inflammatory microenvironment, thereby perpetuating a detrimental feedback loop [47,48].

### Angiopathy

Angiogenesis plays a crucial role in the wound healing process, with the development of CWs being triggered by poor tissue perfusion and reperfusion injury caused by ischemia [49]. In acute wounds, vascular injury restricts oxygen delivery, creating a hypoxic environment around the wound that stimulates cell proliferation and initiates tissue repair. In contrast, prolonged hypoxia in CWs impairs the wound healing process by inhibiting angiogenesis, re-epithelialization, and ECM synthesis [50]. Ischemia and subsequent tissue hypoxia can induce a proinflammatory state, which exacerbates tissue hypoxia by recruiting inflammatory cells with high oxygen consumption to the wound area, leading to tissue necrosis, ulceration, and delayed wound healing.

Prolonged reduction in arterial blood supply results in tissue ischemia and hypoxia, accumulation of metabolites, and, in severe cases, tissue necrosis. In parallel, the structure and function of the microcirculation undergo (micro)alterations. The imbalance of NO content and the decreases in vascular endothelial growth factor (VEGF), nerve growth factor, basic fibroblast growth factor (bFGF), and other angiogenesis-stimulating growth factors contribute to impaired wound angiogenesis [51]. In addition, repeated ischemia–reperfusion injury on the basis of tissue ischemia also affects the formation of difficult-to-heal wounds. Following ischemia–reperfusion injury, inflammatory cells infiltrate tissues in response to chemokine recruitment and release proinflammatory cytokines and oxygen free radicals. The content of nitrous oxide (N_2_O) decreases, resulting in vasoconstriction and inadequate tissue perfusion, thus deteriorating tissue damage [52]. Ischemia and hypoxia can impede proliferation and differentiation of vascular endothelial cells in the wound, hindering the formation of granulation tissue and complicating wound closure [53]. Therefore, reduced local blood supply, delayed wound vascularization, and inhibited angiogenesis all contribute to diminished granulation tissue formation and slow wound healing. It is well documented that promoting local blood supply and improving the ischemic and hypoxic condition of injured tissues can facilitate wound healing. Guan et al. [54] have reported that increasing the local oxygen supply of the wound can promote the survival and migration of keratinocytes and fibroblasts, as well as increase the expression of angiogenic growth factors and angiogenesis in diabetic wounds, while reducing the expression of proinflammatory cytokines, thereby leading to significantly improved wound closure rate. Xiong et al. [55] successfully synthesized a multifunctional hydrogel biomaterial based on M2-Exos (HA@MnO2/fibroblast growth factor-2 [FGF-2]/Exos) to support the healing of diabetic wounds. Local injection of HA@MnO2/FGF-2/Exos can release M2-Exos and FGF-2 to promote angiogenesis and epithelial regeneration, respectively, thus improving wound healing [55].

In addition, in light of the negative impact of oxidative stress and deficient angiogenesis on tissue regeneration, researchers have developed immunomodulatory nanosystems with antioxidative and proangiogenic abilities [56]. These nanosystems aim to improve the beneficial effects of wound healing by addressing these specific challenges. Immunomodulatory nanosystems have been designed with inherent antioxidative and proangiogenic properties, and create a favorable immune-microenvironment that promotes tissue regeneration [7,8]. Moreover, nanosystems can be loaded with antioxidant compounds and proangiogenic agents to enhance their therapeutic potential. Antioxidant molecules such as vitamins C and E, along with natural extracts like resveratrol and curcumin, can be incorporated into these nanosystems for sustained release at the wound site. This sustained release ensures a continuous supply of antioxidants, effectively reducing oxidative stress and fostering a regenerative environment [57]. The targeted delivery and controlled release of antioxidants and proangiogenic agents via immunomodulatory nanosystems offer a promising strategy to address the challenges posed by oxidative stress and deficient angiogenesis in wound healing.

### Neuropathy

The skin features a complex sensory network that detects a variety of chemical, mechanical, and thermal stimuli throughout the body [58]. Simultaneously, the nervous system plays a crucial role in maintaining the delicate balance of the wound inflammatory process, host defense against pathogens, as well as tissue repair. The nervous system rapidly processes information and orchestrates complex defensive behaviors. The immune system mobilizes a variety of specific immune cell populations to eliminate various threats. These 2 systems are closely intertwined to maintain homeostasis in response to tissue injury and infection [59].

During normal wound healing, immune cells release cytokines, including TNF-α, IL-1β, and IL-6. These cytokines interact with receptors on nerve endings in the peripheral nervous system, triggering the generation of action potentials that are transmitted to the brain for pain perception [59,60]. Activated pain receptors locally release neuropeptides and neurotransmitters in the skin, causing vasodilation, increased capillary permeability, and neurogenic inflammation [61]. Skin sensory nerves and neuropeptides play a regulatory role in inflammation, immunity, cell proliferation, and vascular proliferation and participate in wound healing. When the nervous system is damaged, the proliferation of fibroblasts, keratinocytes, and endothelial cells will be weakened, which blocks the process of wound epithelialization during skin healing. Meanwhile, the weakening of neurovascular interaction leads to the obstruction of vascular regeneration. The function of skin neuroimmune function to maintain homeostasis will be reduced. The above factors will cause difficulties in the CW healing. Diabetic foot ulcers, which rank among the most common causes of CWs, are characterized by neuropathy and reduced peripheral sensation [62]. Patients are usually more prone to repeated injuries and secondary infections due to impaired lower limb sensation. Alapure et al. [63] demonstrated severe impairment in wound healing processes, including re-epithelialization and granulation tissue formation, in the presence of denervation, suggesting that damage to cutaneous nerves may inhibit healthy wound healing. Neuropathy and injury also affect endogenous wound repair by regulating stem cell activity. Huang et al. [64] noted that skin denervation could phenocopy the effects of diphtheria toxin-mediated ablation of leucine-rich repeat-containing family of G-protein coupled receptor-6 (LGR6) stem cells in wound healing. They concluded that LGR6 epidermal stem cells interact with nerves to regulate their fate. Thus, sensory nerves can modify the wound healing process by regulating the function of epidermal stem cells [65].

In summary, the sensory nervous system has an essential function in the process of wound healing, regulates wound healing through neuroimmune interactions in response to pathogens, and maintains homeostasis during tissue repair. These regulatory mechanisms are complex and highly dependent on the pathological or inflammatory environment. Investigating the precise molecular mechanism of neuroimmune crosstalk and elucidating the involved pathophysiological processes are crucial for regulating inflammation, tissue repair, and host defense against pathogens. Future investigations are needed to decipher new molecular targets for promoting wound healing. Xiong et al. [7] designed a self-healing and bioadhesive hydrogel to coordinate neurogenesis and angiogenesis simultaneously, leading to an increased efficacy of current diabetic wound therapies. This design puts forward the concept of supportive neurogenesis–angiogenesis crosstalk throughout the whole healing process of CWs. Therefore, promoting nerve regeneration could promote CW healing through the neuroimmunity and neurogenesis–angiogenesis interaction.

### Multiple bacterial infections

Another significant factor contributing to the pathogenesis of CWs is multiple bacterial infections [66]. The presence of wound exudate and necrotic tissue creates a favorable environment for bacterial growth, with the bacterial escape from the host immune response increasing the likelihood of infection. Rahim et al. [67] have documented that CWs harbor various bacterial species, including *Staphylococcus*, *Pseudomonas*, *Corynebacterium*, *Streptococcus*, *anaerobic Cocci*, and *Enterococci*. Among these, the most common wound pathogens are *Pseudomonas aeruginosa* and *Staphylococcus aureus* [68]. In addition, CWs typically hold a more diverse bacterial colonization compared to acute wounds. Proliferating bacteria can lead to wound infection, impair re-epithelialization of wound repair cells, and consequently delay wound healing.

Bacterial infection triggers leukocyte chemotaxis, resulting in the secretion of numerous inflammatory factors, proteases, and ROS, initiating inflammatory cascades and causing excessive and persistent inflammation of the wound. Meanwhile, the abundance of proteases and ROS may degrade growth factors and ECM, hinder cell migration, and delay wound healing. Moreover, bacteria colonize CWs, proliferate, and form clones embedded within a multilayer matrix composed of necrotic tissue and ECM, leading to biofilm formation [69]. Biofilms provide an optimal environment for bacteria to evade immune responses and antibiotics, thus sustaining chronic inflammation in the wound. In addition, bacterial proliferation and the secretion of toxic factors impede the wound healing process [70]. Biofilms also attract activated neutrophils that continuously generate ROS by depleting O_2_ in the microenvironment. However, this oxygen-deprived state fails to eradicate the bacteria, resulting in delayed or halted wound healing. *Staphylococcus aureus* biofilms release leukocidins, inducing extracellular trap formation and evading neutrophil-mediated killing [71]. Finally, bacterial infection leads to a decrease in anabolic hormones, an increase in catabolic hormones, and a high metabolic state or even sepsis, which can complicate wound healing even further [72].

In summary, most CWs are complicated by multiple bacterial infections, fostering the formation of biofilms and sustaining chronic inflammation. Bacterial proliferation and the secretion of toxic factors impede the wound healing process. Greater attention has been directed toward understanding the role of wound microflora in the wound healing process, with modulation of wound microflora potentially offering a promising avenue to promote wound healing. For instance, Shaky et al. [73] synthesized ultrafine silver nanoparticles (AgNPs) with a size of approximately 2 nm using water-soluble and biocompatible γ-cyclodextrin metal–organic frameworks (CD-MOFs). These AgNPs were easily dispersed in an aqueous medium and showed effective bacterial inhibition, further promoting the synergistic effect of wound healing and antibacterial effect. Tong et al. [74] successfully developed PB@PDA@Ag nanocomplexes that eradicated multidrug-resistant bacteria and accelerated wound healing in a methicillin-resistant *Staphylococcus aureus*-infected diabetic model with the aid of laser irradiation.

## Characteristics of CW Immune Microenvironment

**Fig. 3.**
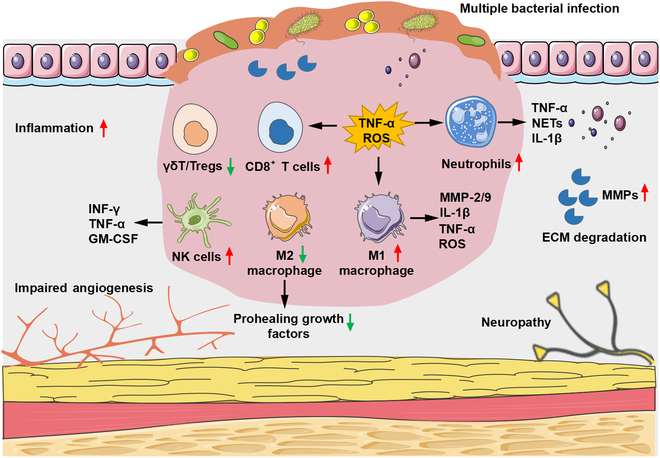
Characteristics of the immune microenvironment in CWs. Dysregulation of M1/M2 in CWs. M1 macrophage infiltration increased and secreted a variety of proinflammatory cytokines into the wound, such as TNF-α, IL-1β, and ROS. Persistent high levels of neutrophils lead to increased protease secretion, ECM, and cell membrane damage. The expression of effector produced by anti-inflammatory T cell subsets such as (γδ) T cells and Tregs decreased. NK cells promote the secretion of proinflammatory cytokines, such as IFN-γ, TNF-α, and GM-CSF.

Wound healing is a complex process involving a wide array of immune cells and molecular factors [75]. In the early stages of skin wound healing, the immune response is essential for clearing pathogens. However, during the formation of CWs, dysfunctional immune cells, including macrophages and neutrophils, contribute to a persistent (hyper)inflammatory microenvironment. As a result, a local immune milieu develops that interferes with the healthy wound healing program. The main roots for delayed wound repair in CWs include increased inflammatory responses caused by the continuous local infiltration of macrophages, T cells, and other immune cells as well as the diminished aggregation and activation of acquired immune cells (Fig. 3) [76]. Therapeutically, it is essential to remodel the immune microenvironment in chronic skin wounds by suppressing the inflammatory response and reducing cytokine production. Therefore, coordinated regulation of the immune microenvironment, innate immunity, and adaptive immunity holds the potential for achieving more favorable clinical-therapeutic outcomes. A comprehensive understanding of the underlying cellular-molecular machinery can provide valuable insights for the development of targeted clinical interventions to promote wound repair [77].

### Macrophages

Macrophages hold a key role in the development of CWs, mainly through their phagocytic and secretory functions [78]. In principle, there are 2 main subtypes of macrophages: proinflammatory macrophages (M1) and anti-inflammatory macrophages (M2). M1 macrophages, also known as classically activated macrophages, are activated in response to pathogen invasion. They release effector molecules (ROS and active nitrogen mediators) and various inflammatory factors (IL-1β, TNF-α, and IL-6) that contribute to eliminating microorganisms which play a pivotal role in the early stages of wound healing. Conversely, M2 macrophages are alternately activated macrophages and secrete anti-inflammatory factors (IL-10), various ECM proteins (fibronectin, TGF-β, and inducible gene-H3), and a wide series of angiogenic factors (TGF-β1, platelet-derived growth factor-B, hepatocyte growth factor, and insulin-like growth factor-1) to promote cell proliferation, collagen deposition, and angiogenesis. By exerting anti-inflammatory effects, M2 macrophages contribute to wound healing and tissue repair [79].

In the normal process of wound healing, M1 macrophages phagocytose and eliminate foreign materials and necrotic cells, while M2 macrophages regulate wound repair by releasing various cytokines [80]. However, in CWs, there is an increased infiltration of M1 macrophages that secrete a plethora of proinflammatory cytokines, including TNF, IL-6, and inducible nitric oxide synthase. Elevated levels of these inflammatory mediators lead to increased recruitment of immune cells, thereby intensifying and maintaining inflammation within the wound and causing local tissue damage [78]. Additionally, imbalanced regulation of M1/M2 macrophages is implicated in impaired wound healing. In fact, diabetic wounds showed depolarization and persistence of M1 macrophages [22], suggesting that the hyperglycemic state was conducive to the persistence of the proinflammatory M1 macrophage phenotype in the wound. Efferocytosis, the process by which macrophages switch from M1 to M2 phenotype, is disrupted in CWs. This interference negatively affects the phagocytic capacity of macrophages and induces an altered ratio of proinflammatory/anti-inflammatory cytokines, ultimately compromising wound healing [81].

Briefly, macrophages play a central role during different stages of wound repair. Dysregulation of macrophage phenotypic transformation and impaired efferocytosis can disrupt the normal wound healing process, thus contributing to the development of CWs. One of the key mechanisms of CW treatment is to regulate M1/M2 phenotype balance. Hauck et al. [82] used collagen/hyaluronic acid-based hydrogels to release sulfuric hyaluronic acid and reduce the activity of inflammatory macrophages, which could improve wound healing in diabetic mice. Tu et al. [83] designed and prepared nanozymes by crosslinking hydrophilic poly (PEGMA-co-GMA-co-AAm) polymers with hyperbranched poly-L-lysine-modified manganese dioxide (MnO_2_). The nanozymes demonstrated the ability to promote wound healing by reducing inflammation levels, neutrophil infiltration, and enhancing M2 macrophage polarization. Nguyen et al. [84] showed that promoting macrophage polarization toward M2 phenotype could improve angiogenesis in diabetic wounds.

### Neutrophils

Neutrophils rapidly accumulate in injured tissue to counter microbial invasion through their bactericidal effects. Additionally, neutrophils are associated with increased inflammation and exert a substantial impact on wound healing [62]. Indeed, the impaired wound healing process in CWs is closely linked to neutrophil dysfunction, particularly factors such as excessive neutrophil activity, disrupted apoptosis, and the overproduction of NETs.

In CWs, an excessive recruitment and retention of neutrophils occur. Sustained high levels of neutrophils result in elevated concentrations of toxic compounds and a proinflammatory microenvironment [62]. The excessive production of neutrophil-derived ROS and increased secretion of proteases contribute to ECM and cell membrane damage and impair vascular processes and blood flow, ultimately manifesting as the characteristic features of nonhealing wounds [18]. Furthermore, delayed neutrophil apoptosis in CWs results in persistent activation of inflammatory cells and release of inflammatory mediators, thereby prolonging inflammation. The clearance of neutrophils begins with apoptosis or necrosis, followed by phagocytosis through macrophages [85]. Via efferocytosis, wound-resident macrophages transition into a tissue remodeling state [86]. Liu et al. [87] reported that, in a diabetic skin wound model, FasL-Fas signaling-induced neutrophil apoptosis facilitated timely regression of inflammation. Subsequently, apoptotic neutrophils were cleared by macrophages, promoting the anti-inflammatory transformation of macrophages and driving regeneration to promote wound healing. NETs are present in high numbers in CWs, carrying neutrophil elastase (NE) and MMP-9 to hydrolyze ECM [88]. The cytotoxicity of NETs can also damage epithelial and endothelial cells, impeding the proliferation of wound keratinocytes and causing delayed wound healing. The process by which neutrophils initiate NETs production is called NETosis. NETosis contributes to delayed wound healing, while inhibiting NETosis accelerates the repair process [89]. Wong et al. [20] demonstrated that disrupting DNase 1 in NETs could expedite wound healing in diabetic and normal glycemic wild-type mice. Inhibiting NETosis or eliminating NETs improved wound healing and reduced NET-driven chronic inflammation in diabetes.

### T cells

T lymphocytes, or T cells, are also widely considered key players in the wound healing process. In fact, following skin injury, T lymphocytes begin to migrate to the wound, reaching a peak at 7 d after injury and gradually decreasing thereafter. The distribution of T lymphocytes within the wound varies at different stages of the wound healing process. T cells can be subcategorized into regulatory T cells (Tregs), gamma delta (γδ) T cells, CD4^+^ T cells, and CD8^+^ T cells according to their specific functionality during wound healing. Tregs represent a subset of anti-inflammatory T cells that play an important role in orchestrating inflammation in the body [90]. Tregs also promote the secretion of anti-inflammatory factors by regulating the function of neutrophils and macrophages, thereby maintaining the balance between immune homeostasis and inflammation and facilitating the process of tissue healing [91,92]. Moreau et al. [93] reported that skin Tregs interact with their tissue environment through transcriptional regulation, influencing epithelial cell biology by upregulating the expression of integrin and TGF-β pathway genes. In addition to their immune regulatory role, the dynamic regulation of Tregs in angiogenesis has gained significant attention [94]. Betto et al. [95] reported that the accumulation of VEGFR1^+^CXCR4^+^Foxp3^+^ Tregs in ulcer tissues could exert a crucial role in tissue healing and angiogenesis through the VEGFR1-TK signaling pathway. Currently, Treg-based cell therapy is being investigated in clinical trials for the treatment of autoimmune diseases, transplant rejection, and graft-versus-host disease, offering promising prospects for tissue repair and regeneration in the future [96].

Gamma delta (γδ) T cells represent a distinct and conserved lymphocyte population with major functions in immune responses and immunopathologies [97]. Xu et al. [98] demonstrated increased levels of effector protein and mRNA expression produced by γδ T cells in acute wound healing model, while their expression was reduced in CW repair models. These findings suggest that γδ T cells are vital for wound healing and exert an important role in inflammatory response. Furthermore, Liu et al. [99] also found that defects in dermal Vγ4^+^ gamma delta T cells (Vγ4^+^γδ) may be an important mechanism contributing to delayed wound healing in diabetic mice. Cytotoxic T cells (CD8^+^ T) and T helper cells (CD4^+^ T) are 2 additional T cell subcategories that significantly contribute to tissue regeneration [100]. In the process of skin regeneration, CD4^+^ and CD8^+^ T cells accumulate in the wounds and secrete a myriad of cytokines that regulate the functions of macrophages and fibroblasts. Davis et al. [101] reported that in a rat wound model with reduced CD4^+^ T cells, the wound’s strength, resilience, and toughness decreased significantly. Notably, in a rat wound model with decreased CD8^+^ T cells, these mechanical properties (i.e., strength, resilience, and toughness) were significantly increased. These experimental observations imply that these 2 subgroups may play opposing roles in mediating wound regeneration.

### Natural killer cells, dendritic cells, and other cells

Natural killer (NK) cells are important immune cells in the body with diverse functions, including antitumor, antiviral, antibacterial, anti-infection, and immune regulation [102,103]. Upon activation, NK cells synthesize and secrete a variety of growth factors and cytokines that affect the wound healing process. NK cells accumulate at the site of skin wounds, typically peaking around 5 d after injury. In wound environments, NK cells predominantly exhibit mature phenotypes characterized by CD11b^+^CD27^−^ and NKG2A^+^NKG2D^−^ expressions. They also express LY49I and proinflammatory cytokines, including interferon-γ (IFN-γ), TNF-α, granulocytic macrophage colony-stimulating factor (GM-CSF), and IL-1β [104]. IFN-γ and GM-CSF can activate macrophages toward M1 polarization [18]. Silva et al. [104] reported that a reduction in NK cell presence in the wound led to decreased expression of IFN-γ, TNF-α, IL-1β, and other proinflammatory cytokines without affecting the aggregation of neutrophils or macrophages. Sobecki et al. [105] noted that the loss of hypoxia-inducible factor-1α in NK cells reduced the release of IFN-γ and GM-CSF, resulting in accelerated wound healing but diminished defense against bacterial infections. This evidence indicates that NK cells impair skin wound healing through the production of proinflammatory cytokines. Therefore, the depletion of NK cells may enhance epithelial cell regeneration, promote collagen deposition, and improve the rate of wound healing but may also increase the risk of bacterial skin infections.

Dendritic cells (DCs) are considered highly efficient professional antigen-presenting cells in the body. They play a critical role in regulating immune responses by uptaking, processing, and presenting antigens, thereby initiating T cell-mediated immune responses [106]. DCs are also capable of synthesizing and secreting a wide range of cytokines, contributing to the maintenance of tissue homeostasis through the regulation of innate and adaptive immunity [107]. Albeit the exact role of DCs in skin healing and regeneration still remains elusive, recent studies have highlighted their role in tissue repair: Maschalidi et al. [4] reported that high expression of Slc7a11 in DCs within the wound skin of diabetic mice. Targeting Slc7a11 can promote the release of TGF-β family member GDF15 from efferocytic DCs, promoting wound healing. Vinish et al. [108] discussed the role of DCs in regulating the healing response after burn wounds. Their findings revealed that the wounds in DC-deficient mice exhibited decreased levels of TGF-β1 and significantly inhibited the formation of CD31^+^ vessels, thereby affecting early cell proliferation, granulation tissue formation, and delayed wound closure. In contrast, DC promotion significantly accelerated early wound closure. These insights suggest that DCs may play a crucial role in expediting early wound healing, with the enhancement of their function serving as a potential therapeutic intervention to promote CW healing.

In conclusion, wound healing is a complex process involving various immune cells and molecules, each of which play distinct roles during different stages of CW healing. Their effect on wound healing is contingent upon their subtype and is further modulated by local signals within the microenvironment. Gaining a deeper understanding of the roles of these immune cells in healing different types of injury is crucial for identifying potential therapeutic targets. Consequently, strategies aimed at regulating the wound immune response and remodeling the local inflammatory microenvironment have emerged as promising approaches for promoting wound healing. These strategies involve the regulation of macrophage polarization, promotion of T cell differentiation into helper T cells, and stimulation of proinflammatory activation markers in DCs to enhance, regulate or inhibit related immune responses. Future research efforts are needed to decipher the full potential of immune cells and immune microenvironments for wound healing by combining knowledge from different injury models and underlying clinical diseases.

## Applications of Immunomodulatory Nanosystems in CW Treatments

Currently, various strategies aimed at targeting the pathogenic immune microenvironment have been developed to mitigate the deterioration of CWs. However, the effectiveness of these strategies falls short of expectations. The complexity of the immune microenvironment in CWs is widely recognized as a crucial factor affecting the healing process. Immunomodulatory nanosystems have emerged as a promising approach for improving wound repair and regeneration due to their excellent physicochemical properties, efficient drug-loading capacity, and favorable biocompatibility [15]. Over the past decade, a wide array of organic, inorganic, and organic–inorganic hybrid nanosystems have been employed in the development of advanced wound dressings, serving as effective drug delivery tools that beneficially modulate the immune microenvironment in CWs. Mechanistically, these versatile nanosystems exert their immunomodulatory roles through 3 main mechanisms: (a) stimulation of M2 macrophage polarization, (b) anti-inflammatory actions, and (c) ROS-scavenging (Table).

**Table.** Nanosystem-based strategies for immunomodulation in CWs.

Mechanisms	Strategies	Example	Details
Induction of M2 macrophage polarization	Reprogram macrophage phenotype	Bioactive glass (BG) [145]	BG directly induces macrophages toward M2 polarization
	Delivery of therapeutic gas	NO@HKUST-1 [120]	Nitric oxide (NO) induces macrophages from M1 to M2 via activation of NO/vasodilator-stimulated phosphoprotein signaling
	Delivery of EVs	G-sEVs^DM^ [7]	DM released from the G-sEVs^DM^ drives macrophage phenotype switch from M1 to M2
	Delivery of stem cells	Fibrous scaffolds encapsulated with adipose-derived mesenchymal stem cells (AD-MSCs) [146]	The released AD-MSCs drive macrophages toward M2 polarization
	Delivery of cytokines	BCL@MMSNPs-SS-CD-NW [147]	Baicalein (BCL) targets macrophages and induce M2 polarization
	Gene therapy	AMPC@siTNF-α [148]	The small interfering RNA released from NPs suppress M1 polarization and induce M2 polarization
Anti-inflammation	Self-activity of NPs	Multifunctional bioactive glass-ceramic nanodrug (BBGN-Mo) [149]	The BBGN-Mo exhibits excellent anti-inflammatory effect
	Delivery of EVs	Poly (ethylene glycol) hydrogels (Exogels) [25]	The macrophage-derived EVs can promote angiogenesis and suppress inflammation
	Delivery of stem cells	PLGA@IL-8/ADM system [26]	This system provides exogenous MSCs for anti-inflammation
	Delivery of cytokines	PLGA-curcumin NPs [150]	NPs exhibit anti-inflammatory effects via release of curcumin
	Delivery of antioxidants	Mitochondria-targeted hybrid nanozymes [151]	The artificial cascade nanozyme possesses superoxide dismutase- and catalase-like activities as well as anti-inflammation
	Delivery of therapeutic gas	DNase-CO@MPDA NPs [152]	The released carbon monoxide (CO) exhibits robust anti-inflammatory activity
	Gene therapy	miR-223-loaded hyaluronic acid NPs [125]	The released miR-223 exhibits anti-inflammation via harness macrophage polarization
ROS scavenge	Antioxidant NPs	Ultrasmall Cu_5.4_O nanoparticles (Cu_5.4_O USNPs) [153]	Cu_5.4_O USNPs with multiple enzyme-mimicking and broad-spectrum ROS scavenging ability
	Delivery of cytokines	Polydopamine NPs (PDA NPs) [154]	PDA degrades ROS and generates oxygen
	Delivery of antioxidants	PDA/PUE/FA NPs [155]	The released ferulic acid (FA) exhibits excellent antioxidant activity
	Delivery of therapeutic gas	Multifunctional nanosystem for delivery of gaseous hydrogen [156]	Hydrogen exhibits ROS-scavenging ability via activation of Nrf2 signaling
	Gene therapy	MS-CeO_2_ nanocomposites [157]	The nano-miR129 released from the composites can significantly enhance anti-ROS efficiency

In fabrication of immunomodulatory nanosystems for wound healing, researchers are actively investigating the clearance mechanisms to ensure their long-term biocompatibility. Several factors are taken into consideration, such as the size, surface properties, and composition of the nanosystems [29]. Nanosystems can be engineered to be biodegradable, allowing them to gradually degrade and be removed or eliminated from the body. Biodegradation can occur through various mechanisms, including enzymatic degradation or breakdown by the body’s natural processes [109]. For example, nanosystems composed of biocompatible polymers like polylactic acid or poly(lactic-co-glycolic acid) (PLGA) can be designed to degrade over time into smaller fragments that can be cleared by the body’s excretory system [110].

In addition to biodegradation, nanosystems can also be designed with surface modifications or coatings to improve their biocompatibility and facilitate their clearance. Surface modifications can influence interactions with biological components, such as proteins or cells, potentially affecting their recognition and uptake by the immune system or clearance organs [111]. To assess the long-term clearance mechanism of nanosystems, researchers conduct comprehensive studies using in vitro and in vivo models. These studies investigate the fate of nanosystems over extended periods, tracking their distribution, metabolism, and elimination from the body [112]. Advanced examinations such as imaging, biodistribution analysis, and histological examination help researchers monitor the behavior of nanosystems in different organs and tissues [112]. It is worth noting that the clearance mechanism can vary depending on the specific nanosystem design, route of administration, and target tissue. Therefore, it is crucial to consider these factors and conduct rigorous preclinical and clinical evaluations to ensure the safety and biocompatibility of nanosystems intended for wound healing applications.

Functionally, immunomodulatory nanosystems can promote hemostasis by enhancing blood clotting mechanisms. They can be designed to carry specific molecules, such as clotting factors or platelet-binding peptides, which facilitate the formation and stabilization of blood clots at the wound site. By promoting clotting, these nanosystems can help control bleeding and prevent further tissue damage, enabling the wound healing process to begin. In this section, we comprehensively summarize the advancements in nanosystem-based strategies for modulating the immune microenvironment in CWs and discuss limitations associated with these functional nanosystems.

### Organic immunomodulatory nanosystems in CW treatments

Due to the uncomplicated synthesis process, versatile functionality, and excellent biocompatibility, organic immunomodulatory nanosystems are of increasing popularity in the management of CWs. Recent evidence has shown that certain polymeric nanostructures can effectively reverse the immune microenvironment while promoting angiogenesis and re-epithelialization in CWs [113,114]. For instance, Sun et al. [115] introduced novel ECM-loading nanofibrous scaffolds, mimicking the dynamic transition of native ECM and modulating the immune microenvironment in CWs. Specifically, fibrinogen and collagen I, key ECM proteins, were incorporated into the nanosystems to replicate the sequential appearance of these proteins during the wound healing process. The results demonstrated that the ECM-biomimetic coaxial nanofibrous scaffold effectively switched macrophage phenotypes from M1 to M2, thereby significantly facilitating the wound healing process. Similarly, Liu et al. [116] introduced an absorbable nanofibrous hydrogel for promoting diabetic wound healing via synergistic modulation of the immune microenvironment. The grafted thioethers on hyaluronic acid consumed excessive ROS during the early inflammation stage, while hyaluronic acid induced the transformation of recruited M1 macrophages toward the M2 phenotype, creating a favorable immune microenvironment for wound healing.

**Fig. 4.**
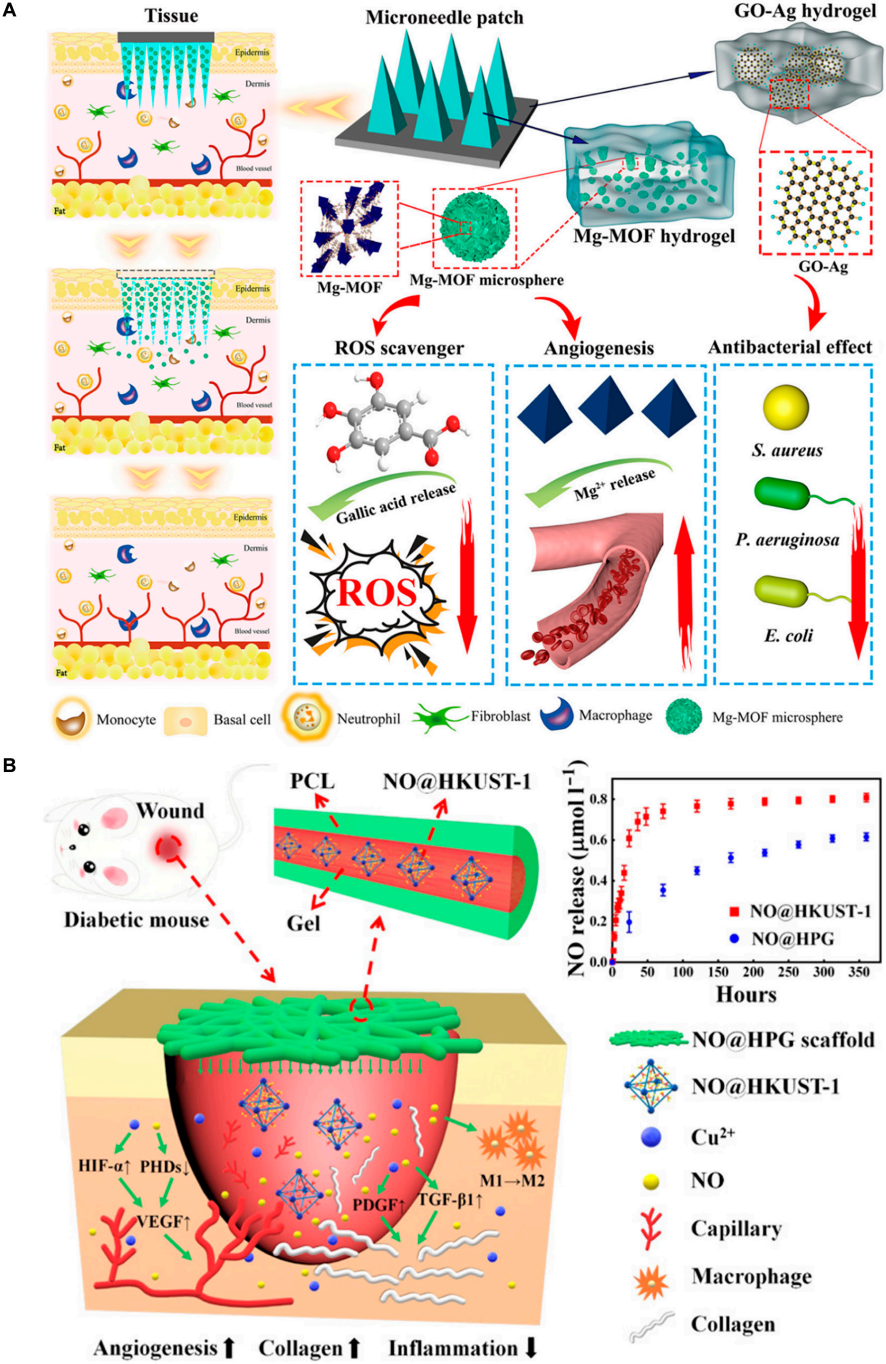
(A) Schematic illustration of MN-MOF-GO-Ag for accelerating diabetic wound healing. Reproduced with permission from [117]. Copyright (2021) American Chemical Society. (B) Schematic illustration of the copper-based MOF scaffold promotes the healing of diabetic wound. Reproduced with permission from [120]. Copyright (2020) American Chemical Society. HIF-1α, hypoxia-inducible factor-1α; PCL, polycaprolactone; PHD, proline hydroxylase.

Metal–organic frameworks (MOFs) may also serve as suitable immunomodulatory nanosystems, particularly in promoting CW healing [117,118]. For example, Wan et al. [119] introduced a zeolitic imidazolate framework-8 nanosystem (Mn-ZIF-8), with enzymatic activity modulating the immune microenvironment in CWs. Structurally, Mn-ZIF-8 features the coexistence of Mn^2+^/Mn^4+^ to endow the nanosystem with enzymatic activity, combating the oxidative microenvironment. In addition to releasing zinc ions, this nanosystem exhibited remarkable antibacterial functionality and remodeled inflammatory immunity by modulating macrophage polarization, thus improving overactivated inflammation and promoting the healing of bacteria-infected CWs. Yin et al. [117] developed a magnesium organic-framework-based microneedle patch (MN-MOF-GO-Ag), which slowly releases Mg^2+^ and gallic acid in the deep layer of the dermis. The multifunctional MN-MOF-GO-Ag resulted in enhanced cell proliferation and antioxidant and antibacterial activities in vitro as well as enhancement of wound healing in vivo (Fig. 4A). Zhang et al. [120] utilized copper-based MOF as a NO-loading vehicle to promote endothelial cell growth and significantly improve the angiogenesis, collagen deposition, as well as anti-inflammatory property in diabetic wound (Fig. 4B). Xia et al. [121] also reported a novel nanosystem (ZIF-8@ Rutin) synthesized by loading Rutin into ZIF-8, which effectively reshaped the immune microenvironment of chronic infected wounds. The encapsulated Rutin provided this nanosystem with immunomodulatory and antioxidant abilities to regulate macrophage polarization.

Moreover, nanosystems also play a crucial role in protecting loaded drugs from degradation while exerting a regulatory function through the controlled release of immunomodulatory agents. Currently, a mounting body of evidence points to the suitable utilization of nanosystems as delivery vehicles for the sustained release of immunomodulatory molecules, aiming to mediate the immune microenvironment in CWs and promote wound healing [122,123]. For instance, Gauthier et al. [124] developed a functional liposome capable of controlled release of dexamethasone to macrophages, thereby providing an anti-inflammatory and prohealing microenvironment for wound repair and regeneration. Saleh et al. [125] introduced nanosystems loaded with microRNA, which acted as local immunomodulatory agents by gradually releasing a miR-223 mimic. Specifically, the released miR-223 effectively guided tissue macrophages toward an anti-inflammatory phenotype polarization and enhanced angiogenesis at the wound site.

Of note, there are also some disadvantages specific to organic immunomodulatory nanosystems in CW treatments. First, organic nanosystems may have limited stability and a shorter shelf life compared to inorganic counterparts. They can be susceptible to degradation over time, which may affect their therapeutic efficacy. Ensuring long-term stability and maintaining the desired properties of organic nanosystems during storage and transportation can be challenging. Second, although biodegradation is a desired feature of organic nanosystems, the rate of degradation can vary and may not always align with the intended duration of treatment. It is crucial to strike a balance between achieving controlled release of therapeutic agents and maintaining structural integrity during the required period. Third, organic nanosystems may have limited loading capacities for therapeutic agents due to their specific compositions and structures. This limitation can restrict the amount of drug or bioactive molecules that can be incorporated into the nanosystems. Moreover, some organic nanosystems may still raise concerns regarding biocompatibility, especially when they are derived from natural sources. The potential for immune responses or allergic reactions to the organic components should be carefully assessed. In addition, organic nanosystems may face regulatory hurdles due to their unique characteristics. They may require additional testing to assess their safety, efficacy, and potential long-term effects.

### Inorganic nanosystems as modulators of the microenvironment

**Fig. 5.**
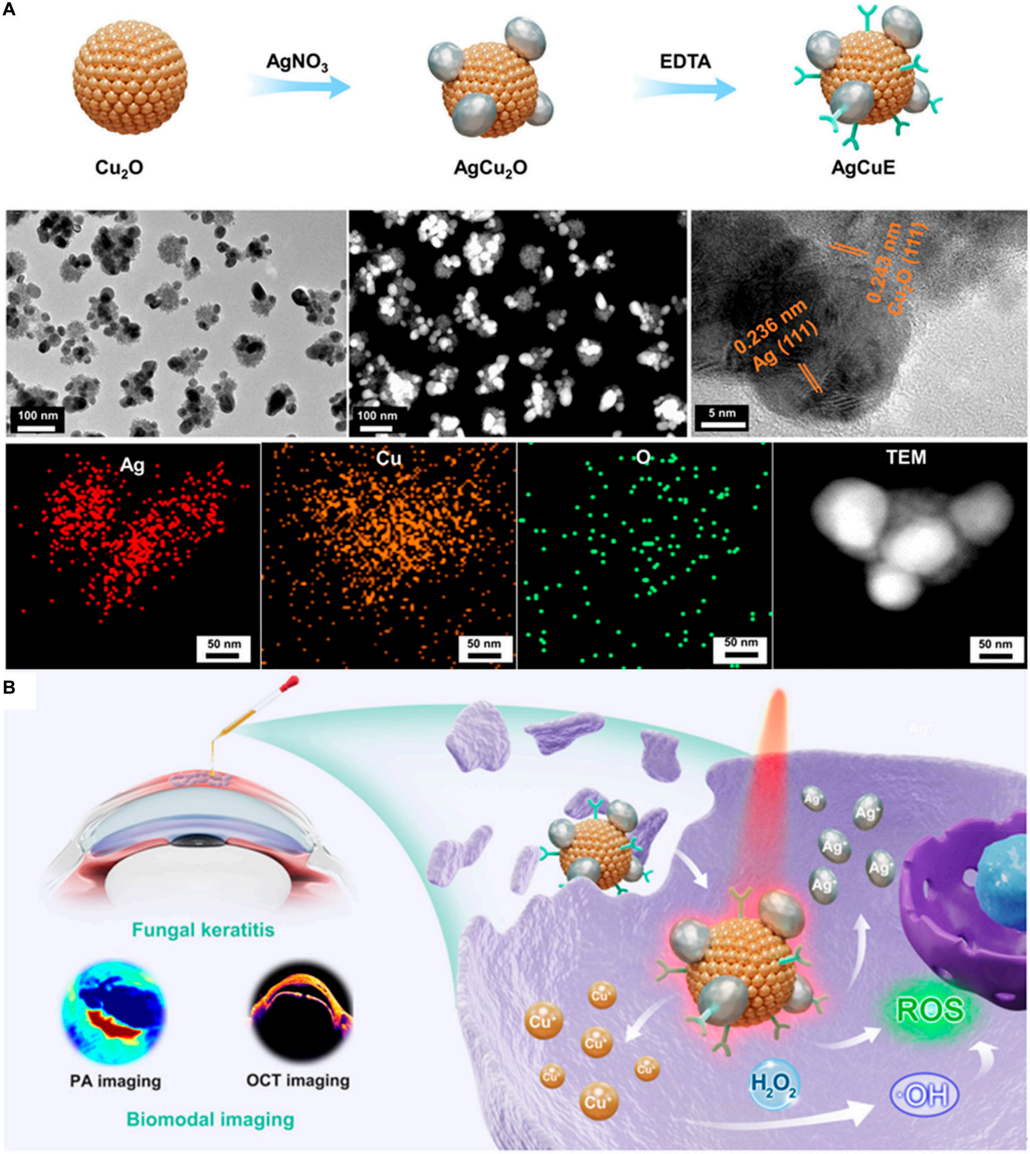
(A) Schematic diagram of AgCuE NPs for fungal keratitis treatment and representative images of transmission electron microscopy (TEM) and X-ray diffraction (XRD) detection. (B) Schematic illustration of the underlying mechanisms of AgCuE NPs in the treatment of fungal keratitis. (A) and (B) were reproduced with permission from [130]. Copyright (2022) American Chemical Society. OCT, optical coherence tomography; PA, photoacoustic.

Due to their superior stability, potent antibacterial activity, and excellent drug controlled-release function, inorganic nanosystems can be widely used for modulating the immune microenvironment and promoting wound healing [126]. In recent years, the field of nanotechnology has witnessed substantial advancements, leading to the development of various inorganic nanosystems for innovative treatments in CWs. Examples of such nanosystems include silver (Ag), cerium (Ce), copper (Cu), graphene oxide (GO), and black phosphorus [127]. Notably, AgNPs serve as a representative example, as they exhibit immunoregulatory properties and can activate the inflammasome, thereby inducing the production and release of neutrophils and NETs to eliminate bacterial infections [128]. Emerging evidence suggests that AgNPs encapsulated with polysaccharide riclin offer antibacterial and anti-inflammatory effects, thereby accelerating CW healing [129]. The beneficial impact on wound healing can be mainly attributed to the significant reduction in proinflammatory cytokines (e.g., IL-1β, IL-6, TNF-α, etc.) observed with riclin/AgNP treatment. Moreover, AgNPs are also used to control fungal infections through cell wall disruption and immunomodulation. A recent study demonstrated the excellent antifungal properties of ethylenediaminetetraacetic acid (EDTA) modified AgCu_2_O nanoparticles (AgCuE NPs) [130]. The nanosystem effectively eradicated fungi by leveraging a synergistic cascade of ion-released chemotherapy, chemodynamic therapy, photodynamic therapy, and mild photothermal therapy. In vivo, AgCuE NPs could contribute to a supportive microenvironment for tissue regeneration, with promising results in animal models of fungal keratitis (Fig. 5).

Furthermore, ceria NPs exhibit enzymatic activity and the ability to scavenge excessive ROS, allowing them to regulate the immune microenvironment of CWs. A recent study presented the development of glucose/ROS dual-responsive Ce NPs as nanozymes and immunomodulatory nanosystems for diabetic wound repair [131]. These versatile NPs were constructed through the encapsulation of dual-ligand molecules, alendronic acid (AL) and 2-methylimidazole (HMIM), into a Ce-driven coassembly, resulting in NPs with robust enzymatic functionality. It is worth mentioning that both in vitro and in vivo assays indicated that this nanosystem effectively alleviated oxidative stress damage and provided a supportive immune microenvironment for cell promotion and differentiation. Wu et al. [33] also introduced a mesoporous silicon decorated with ceria nanocrystals (MSN-Ceria) for promoting wound repair. The MSN-Ceria nanosystem showed significant scavenging of excessive ROS, inhibition of fibrotic proliferation, and a notable reduction in persistent inflammation, along with a significant decrease of CD68-positive macrophages.

In addition, nanosystems can also suppress inflammation and promote microcirculatory blood flow by generating localized high temperatures through the photothermal effect of NPs themselves or by carrying a photothermal agent under laser irradiation. Ouyang et al. [132] proposed a novel therapeutic strategy to address the challenges encountered in the treatment of diabetic wounds. Specifically, to address exacerbated inflammation and angiopathy, the researchers developed a smart gel based on black phosphorus with rapid formation and near-infrared light responsiveness. This multifunctional black phosphorus-based gel not only enhanced local angiogenesis but also created a favorable immune microenvironment. Under near-infrared laser (808 nm) irradiation, the gel generated local heat, improving microcirculatory blood flow, bacterial elimination, and inflammation reduction through photothermal therapy.

Notably, some limitations of the application of inorganic nanosystems in wound healing should be emphasized. First of all, the inorganic nanosystems, especially those composed of metals or metal oxides, may raise concerns regarding their biocompatibility and potential toxicity. The interaction of inorganic nanoparticles with biological systems can trigger immune responses and lead to cytotoxic effects. Furthermore, the clearance mechanisms of inorganic nanosystems from the body may vary depending on their size, shape, and surface properties. Larger nanoparticles may have slower clearance rates, leading to potential long-term accumulation in organs or tissues. Third, achieving controlled release of therapeutic agents from inorganic nanosystems can be more challenging compared to organic counterparts. Although inorganic nanosystems can provide sustained release to some extent, the precise control of release kinetics may be more difficult due to their inherent properties. Moreover, inorganic nanosystems may be prone to aggregation or instability under certain conditions, such as changes in pH or ionic strength. Aggregation can impact their performance and hinder their ability to deliver therapeutic agents effectively. Additionally, inorganic nanosystems may face regulatory considerations and require thorough evaluation for safety and efficacy.

### Utilization of organic–inorganic hybrids in CW treatments

**Fig. 6.**
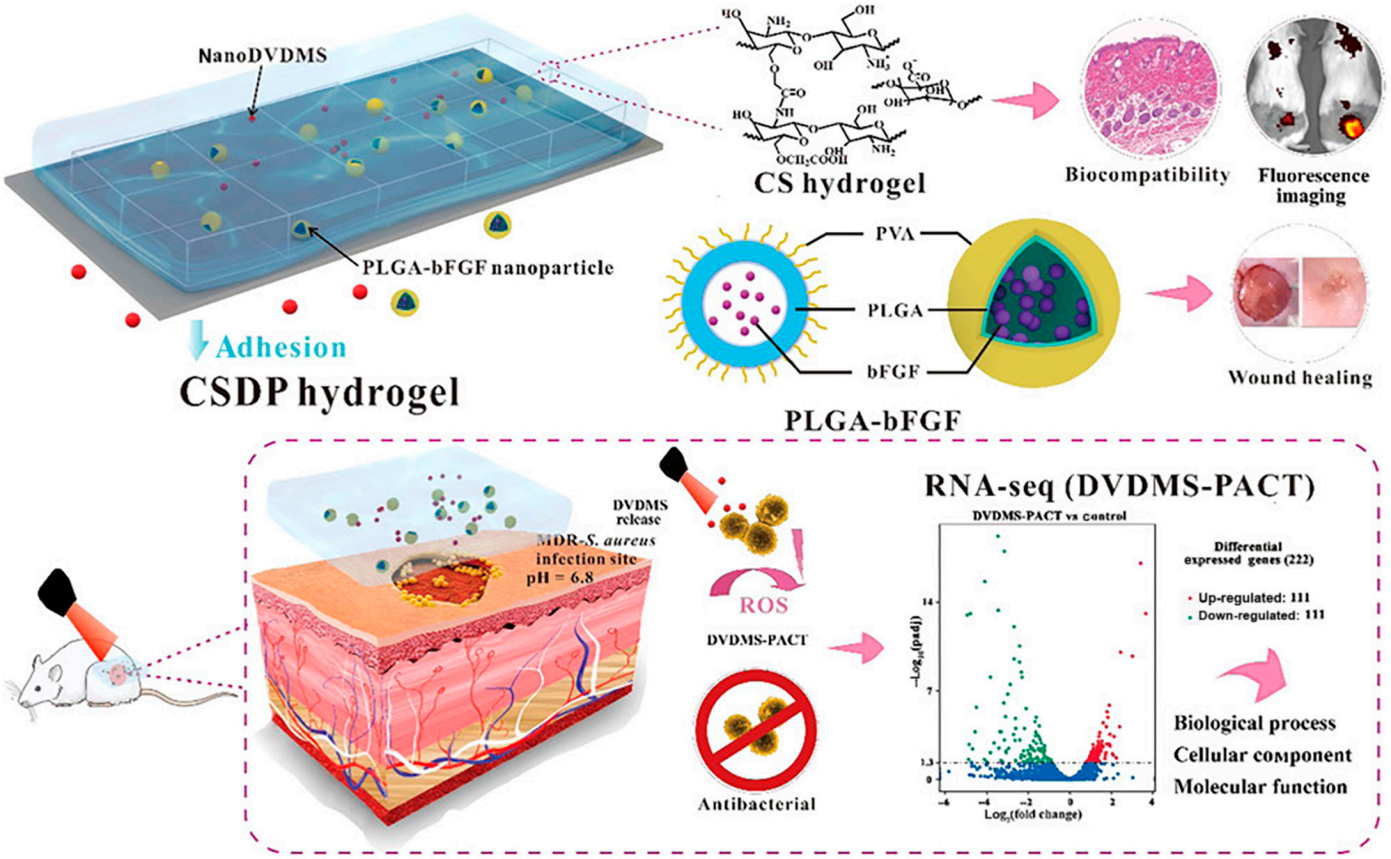
An example of the hybrid nanosystems applicated in CW treatment. The system combines porphyrin photosensitizer sinoporphyrin sodium (DVDMS) and PLGA-encapsulated bFGF nanospheres embedded in carboxymethyl chitosan-sodium alginate to form CSDP hybrid hydrogel. Reproduced with permission from [135]. Copyright (2020) American Chemical Society. RNA-seq, RNA sequencing; PVA, poly(vinyl alcohol); PACT, photodynamic antimicrobial chemotherapy.

Extensive research has established the multifunctional properties of inorganic nanosystems, which include immunomodulatory, antibacterial, and prohealing activities. A series of organic nanomaterials have shown the capacity to induce tissue repair and regeneration by modulating the immune response and promoting ECM deposition. Consequently, various organic–inorganic hybrid nanosystems were developed in the field of biomedicine, particularly for applications in wound repair. Of note, increasing evidence highlights the promising use of these hybrid nanosystems in immune-modulatory tissue engineering for CWs [133,134]. In a recent study, a hydrogel-based nanodelivery system was fabricated with antibacterial activity and skin regeneration function for the treatment of burn infections [135]. The system combines porphyrin photosensitizer sinoporphyrin sodium (DVDMS) and PLGA-encapsulated bFGF nanospheres embedded in carboxymethyl chitosan-sodium alginate to form CSDP hybrid hydrogel. The in vitro results showed that the CSDP nanosystem exhibited excellent antibacterial and antibiofilm activities. In vivo, CSDP was successful in inhibiting bacterial growth and promoting wound healing in a mouse model. The treatment also led to an increase in regenerative factors and a reduction in proinflammatory factors in the burn wounds, indicating its potential as a synergistic treatment for burn infections. This study suggests that CSDP could serve as a promising nanoplatform for the comprehensive treatment of burn infections, combining antibacterial activity with wound healing promotion (Fig. 6).

Organic–inorganic hybrid nanosystems are emerging as a promising group of nanomaterials with significant potential in modulating the immune microenvironment for the treatment of CWs. These nanosystems are multifunctional, including having drug-loading and releasing capabilities, anti-inflammatory effects, antimicrobial properties, and immune response remodeling capabilities [129]. The controlled release of multiple drugs from biomaterials remains a major challenge, and the utilization of nanosystems provides the opportunity to develop nanocomposites with scaffold-forming functions and customizable drug release properties. Mebert et al. [136] introduced a collagen–silica hybrid nanosystem designed for the sustained release of 2 topical antibiotics to eliminate infection in CWs. Structurally, these nanosystems consisted of core–shell silica particles encapsulated with gentamicin sulfate and sodium rifamycin, and they were constructed with type I collagen. Importantly, in vivo results demonstrated the robust immunomodulatory role of this hybrid nanosystem in reshaping the inflammation triggered by infection. Furthermore, in a rabbit model, this nanosystem exhibited excellent controlled dual drug delivery, effectively eliminating wound infection and promoting tissue repair and regeneration. Additionally, to overcome the limited cell wall adhesion function of typical antibacterial Ag nanoparticles, Wang et al. [137] developed mesoporous silica-coated Ag nanocubes loaded with gentamicin. Functionally, the Ag nanocubes with mesoporous silica coating demonstrated potent antibacterial effects, and the presence of intracellular H_2_S in the natural bacterial environment stimulated the release of Ag from the nanospheres, synergistically enhancing the antibacterial activity. Immunohistochemical staining of CD86 in the wounds revealed a significant reduction in proinflammatory M1 macrophages in the nanosystem-treated group compared to other groups. This study underscored the potential of versatile nanosystems in depleting proinflammatory macrophages to facilitate the healing of CWs.

Organic–inorganic hybrid-based immunomodulatory nanosystems offer unique advantages by combining the properties of both organic and inorganic materials. However, they also have some disadvantages in the context of CW treatments. The synthesis and characterization of organic–inorganic hybrid nanosystems can be complex and require specialized techniques. The integration of organic and inorganic components necessitates precise control over the synthesis parameters to achieve the desired hybrid structure and properties. Furthermore, organic and inorganic materials may have different properties, such as surface charges, degradation rates, or interactions with biological systems. Achieving compatibility between these materials within the hybrid nanosystems can be challenging. Third, the biodegradation and long-term stability of organic–inorganic hybrid nanosystems can be complex to predict and control. The degradation rates of organic and inorganic components may differ, potentially leading to changes in the structure and properties of the hybrid nanosystems over time. Moreover, the combination of organic and inorganic components can introduce new challenges in terms of biocompatibility and potential toxicity. The presence of inorganic materials may raise concerns about their cytotoxicity or immune responses. In addition, organic–inorganic hybrid nanosystems may face additional regulatory considerations due to their complex composition and unique properties.

### Engineered extracellular vesicles as regulators of immune microenvironment

Extracellular vesicles (EVs) are nanoscale intercellular messengers carrying a variety of bioactive factors and cytokines, including ribonucleic acid (RNA), proteins, lipids, and deoxyribonucleic acid (DNA). These factors and cytokines can be taken up by recipient cells and are involved in a wide array of biological regulations. Specifically, EVs are able to penetrate tissues, enter the bloodstream, and even cross the blood–brain barrier [138]. With the recent advancements in nanotechnology, engineered EVs with enhanced cell-targeting capabilities and regulatory activities are emerging as multifunctional nanosystems for promoting the healing of CWs, particularly by modulating the immune microenvironment of CWs [139]. Generally, strategies for engineering EVs involve modifications of both donor cells and EVs directly. Recent studies have shown that preconditioning donor cells is a promising strategy to improve immunomodulatory functions and accelerate CW healing through various pretreatments [140]. For instance, Ti et al. [141] introduced an engineered EV derived from lipopolysaccharide-preconditioned mesenchymal stromal cells (LPS pre-Exo) for diabetic wound healing. In vitro results indicated that LPS pre-Exo exhibited a superior function to untreated MSC-derived EVs (un-Exo) in modulating macrophage balance in diabetic wounds. Mechanistically, LPS pre-Exo significantly increased anti-inflammatory cytokine levels and induced M2 macrophage polarization. In a diabetic rat model, in vivo results suggested that LPS pre-Exo substantially facilitated diabetic wound healing by modulating the immune microenvironment and reducing excessive inflammation at the wound site. Similarly, in another study, melatonin (MT)-preconditioned MSCs-derived EVs (MT-Exo) showed enhanced effects on diabetic wound healing by inducing macrophage polarization toward the M2 phenotype [142]. In vitro and in vivo findings indicated that MT-Exo alleviated overactivated inflammatory responses by increasing the M2/M1 macrophage ratio through activation of the phosphatase and tensin homolog/protein kinase B (PTEN/AKT) signaling pathway. Therefore, MT-Exo holds promise as a strategy for facilitating tissue repair and regeneration.

**Fig. 7.**
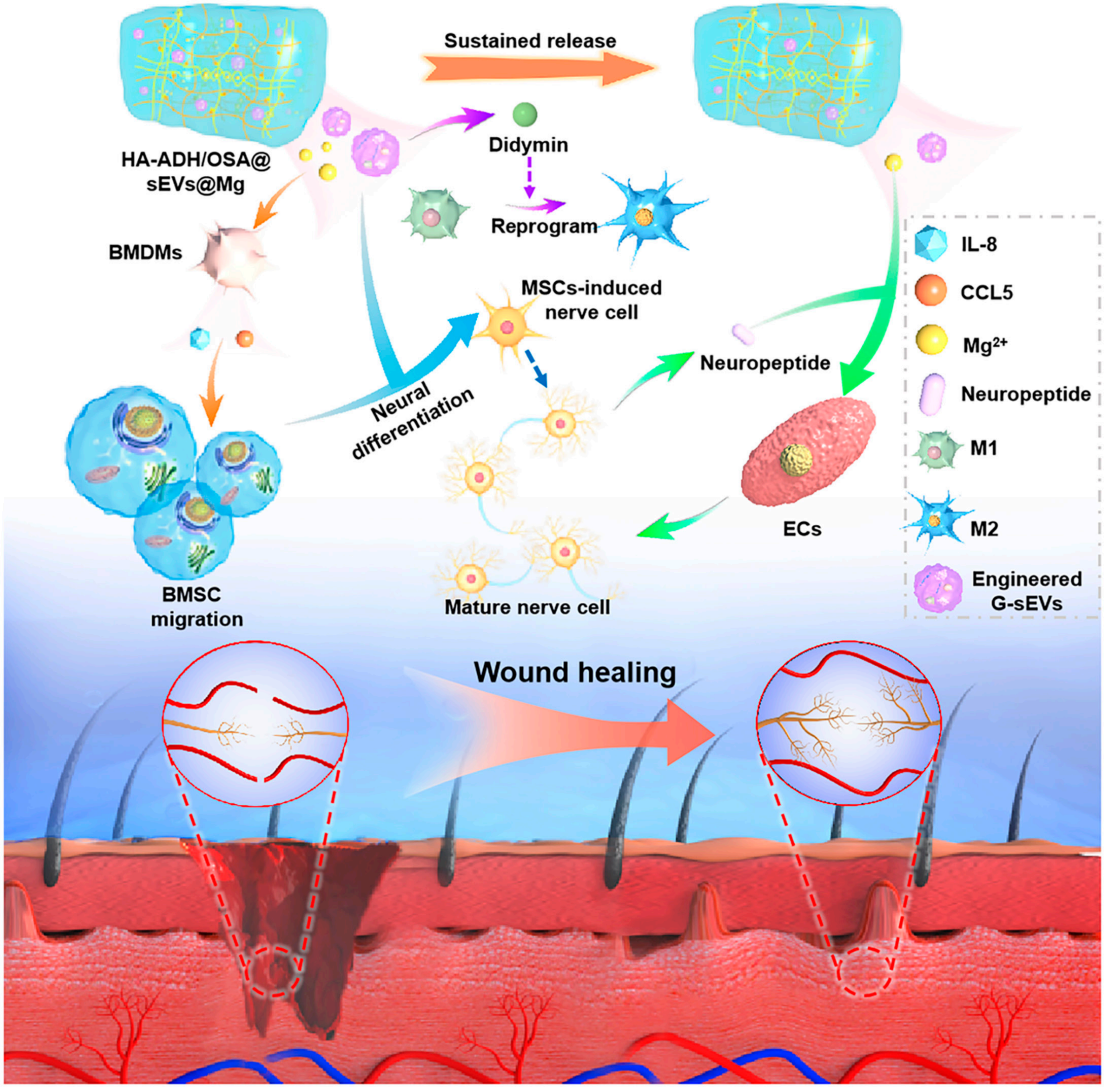
Schematic diagram of the important role of G-Evs^DM^ in the promotion of diabetic wound healing. Reproduced with permission from [7]. Copyright (2023) Wiley. BMDMs, bone marrow-derived macrophages; BMSCs, bone marrow-derived mesenchymal stem cells; ECs, vascular endothelial cells.

In addition to donor cell preconditioning, direct modification of EVs is an important method for obtaining engineered EVs. Advanced loading approaches, such as electroporation, sonication, freeze and thaw cycles, and extrusion, enable efficient encapsulation of bioactive cargos in EVs, thereby enhancing their regulatory activities [143]. In a recent study, the Shen group [144] reported the novel engineering of EVs (E-EVs) collected from TNF-α-treated MSCs under hypoxia, with encapsulation of cationic antimicrobial carbon dots. In vitro, E-EVs demonstrated excellent immunomodulatory function by promoting M2 macrophage polarization and ameliorating excessive inflammation. In vivo results suggested that E-EVs accelerated the healing of infected diabetic wounds and beneficially reversed the immune microenvironment in diabetic wounds. In our previous study, we engineered EVs (G-EVs^DM^) by encapsulating didymin (DM) into EVs derived from ginseng [7]. Through the sustained release of DM, these engineered EVs effectively reprogrammed macrophage phenotypes from M1 toward M2 polarization and provided a beneficial microenvironment for wound repair and tissue regeneration (Fig. 7). The in vivo results implied that the administration of injectable multifunctional hydrogels encapsulated with G-Evs^DM^ resulted in a significant enhancement of wound healing in a diabetic mouse model. Taken together, these studies are a testament to the promising prospects of direct EV modification as a valuable avenue to improve the healing of CWs.

Currently, engineered EVs-based immunomodulatory nanosystems have attracted considerable attention for their potential in CW treatments. However, they also have certain disadvantages that should be considered. Engineering EVs to enhance their immunomodulatory properties can be a complex process. Modifying the cargo, surface composition, or targeting abilities of EVs requires sophisticated engineering techniques. The optimization and reproducibility of these engineering processes can be challenging, potentially limiting the scalability and widespread application of EV-based nanosystems. Furthermore, efficient loading of therapeutic cargo into engineered EVs can be a considerable challenge. Depending on the cargo type and size, achieving optimal loading may require specific strategies or modifications, which can affect the overall manufacturing process and scalability. Moreover, the stability and release kinetics of cargo from engineered EVs need to be carefully considered. Cargo molecules can be sensitive to the harsh extracellular environment or undergo premature release during storage or transportation. In addition, ensuring consistent quality and standardization of engineered EV-based nanosystems is crucial for their safe and effective use in CW treatments. Establishing robust quality control measures, including characterization techniques and standardized protocols, can be challenging due to the complexity and heterogeneity of EV populations.

## Challenges and Perspectives

The field of nanotechnology has made remarkable advancements in both basic research and clinical applications, offering a potential breakthrough for addressing the challenges associated with CW treatment. Immunomodulatory nanosystems, which modulate the immune (micro)environment in CWs, are of increasing popularity. These nanosystems, constructed using multifunctional composite materials, offer the capability to precisely and effectively regulate the local immune microenvironment of CWs to ultimately promote wound healing. Despite their promising potential, several challenges still need to be addressed prior to clinical translation:

1. It is imperative to systematically investigate the safety of nanosystems. When creating innovative nanosystems, the design and preparation must ensure application safety. In this context, improving the targeting specificity, optimizing biocompatibility, and minimizing impacts on both target and nontarget tissues are essential considerations. To achieve mass production and guarantee quality control of nanocarriers, strict manufacturing control conditions are necessary. Ultimately, this has the potential to revolutionize clinical practice, with patients and care providers benefiting from this innovative technology.

2. Local immune dysfunction is one of the major pillars in the complex and intricate healing process in CWs. Although immunomodulatory nanosystems were found to be effective in animal studies, their translation to human skin has been less successful. In fact, rat and human skin defects exhibit distinct healing processes: rodent skin predominantly heals through contraction, whereas human skin heals through epithelial development. Therefore, future investigations are required to elucidate the immunological effects of nanomaterials and their interactions with the human immune system. This is essential for optimizing the shape, size, surface, and physical and chemical properties of nanomaterials.

3. The lack of standardized and reliable models for CWs represents a major challenge. CWs include a variety of different types, such as full-thickness, diabetic, or infected wounds. The majority of research addressing these wounds with immunomodulatory nanosystems has primarily focused on a limited subset of these wound types, potentially leading to biased outcomes or insufficient data supporting treatment. Furthermore, there is a lack of standardization in the evaluation of the wound healing progress after treatment, including changes in wound morphology, biomarker changes at different stages, and scar quality. Establishing standardized production and preparation procedures is pivotal to advancing the clinical application of nanomedicine. Additionally, regulatory controls must be intensified, with a particular focus on harmonizing and improving approval regulations to foster the development of new drug applications, thereby facilitating preclinical research and approval.

4. There is still much work to be done before we can fully rely on nanotechnology to heal CWs, despite these potential benefits. Currently, the use of nanotechnology in commercial wound dressings is primarily restricted to those that incorporate silver nanocrystals as an antibacterial ingredient. The absence of necessary and precise data on the destiny and toxicity of NPs is one of the major problems. This is partly because NPs are complicated and their categorization presents difficulties. Other factors to be taken into account are the design of preclinical and clinical trials as well as the effectiveness of current CW treatments, including those that use nanotechnology. In spite of these problems and difficuties, the interdisciplinary nanoengineering combination of basic medicine and advanced clinical research efforts is expected to largely offer great potential for balancing CW burden.

In summary, as research on immunomodulatory nanosystems for CW treatment progresses from basic science research to clinical applications, it is essential to address the challenges at hand. Systematic investigations into safety, optimization of nanomaterial properties, standardization of wound models, and strengthening of regulatory controls will facilitate the successful translation of nanomedicine into clinical practice.
